# Crystal structure and identification of resonance forms of diethyl 2-(3-oxoiso-1,3-di­hydro­benzo­furan-1-yl­idene)malonate

**DOI:** 10.1107/S2056989017013962

**Published:** 2017-09-29

**Authors:** Mikhail S. Tyumentsev, Mark R. StJ. Foreman, Britt-Marie Steenari, Alexandra M. Z. Slawin

**Affiliations:** a Nuclear Chemistry and Industrial Materials Recycling, Department of Chemistry and Chemical Engineering, Chalmers University of Technology, Gothenburg, Sweden, SE 41296; b School of Chemistry, University of St. Andrews, Purdie Building, North Haugh, St. Andrews, Fife, Scotland, KY16 9ST

**Keywords:** crystal structure, 3-alkyl­idene-3*H*-isobenzo­furan-1-ones, delocalization, enolate

## Abstract

The reaction of diethyl malonate with phthaloyl chloride in aceto­nitrile in the presence of tri­ethyl­amine and magnesium chloride results in the formation of the title compound, diethyl 2-(3-oxo-1,3-di­hydro-2-benzo­furan-1-yl­idene)propane­dioate, C_15_H_14_O_6_. One of the ester groups of the diethyl malonate fragment is almost coplanar with the isobenzo­furan unit, while the plane of the other group is perpendicular to it [dihedral angles = 5.45 (3) and 83.30 (3)°, respectively]. The C—C and C—O distances both in the heterocyclic furan ring and the diethyl malonate fragment are indicative of the dipolar delocalization occurring within the isobenzo­furan unit. This delocalization is likely to be responsible for the unusual inter­molecular O⋯O contact [2.756 (2) Å], established between the O atom of the furan ring and the carbonyl O atom of the diethyl malonate fragment. In the crystal, weak C—H⋯O inter­actions are observed, which link the mol­ecules into [100] chains.

## Abstract 2

The dominant resonance form of the title compound was identified from bond lengths and may correlate with a short intra­molecular O⋯O contact.

## Chemical context   

The structural analysis of diethyl 2-(3-oxoisobenzo­furan-1(3*H*)-yl­idene)malonate (**I**) was undertaken as part of a study into the synthesis of new reagents for the recovery of trivalent lanthanide metal ions by liquid–liquid extraction. We intended to prepare 2,2′-phthaloylbis(*N*,*N*,*N*′,*N*′-tetra­butyl­malon­amide) (**II**), which is similar to the reported earlier 2,2′-[1,2-phenyl­enebis(methyl­ene)]bis­(*N*,*N*,*N*′,*N*′-tetra­butyl­malon­amide) (**III**) (Tyumentsev *et al.*, 2016 ▸), from the respective tetra­ethyl 2,2′-phthaloyldimalonate (**IV**). In turn (**IV**) was to be made by the reaction of diethyl malonate with phthaloyl chloride. It is already known that acid chlorides react with diethyl malonate when treated with a combination of tri­ethyl­amine and a mild Lewis acid (magnesium chloride) in aceto­nitrile (Rathke & Cowan, 1985 ▸). Instead of (**IV**), an organic product, which contained two ethyl groups in different electronic environments, was obtained in this reaction. Crystals of this compound were grown and examined with single-crystal X-ray diffractometry, and the product was found to be the title compound, (**I**). The formation of (**I**) can be rationalized by the nucleophilic attack of the oxygen atom (in an enol form) of the keto-di­ethyl­malonate group on the carbon atom of the unreacted acid chloride group. The mechanism of the formation of (**I**) was proposed by Naik *et al.* (1988 ▸), who obtained this compound by another reaction.
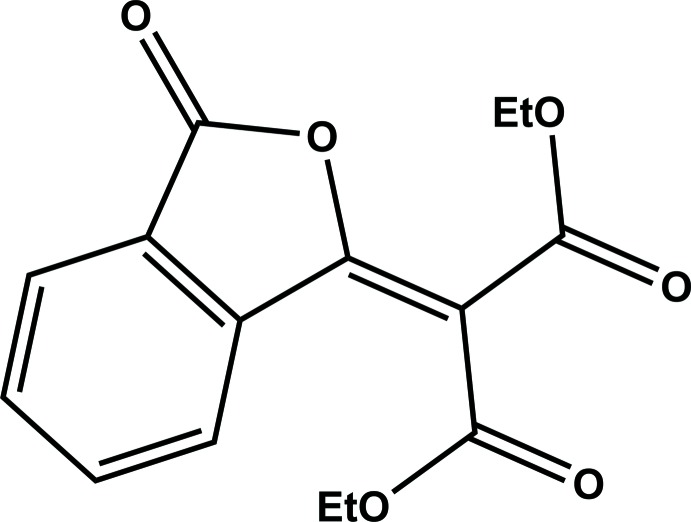


## Structural commentary   

**Figure 1:**
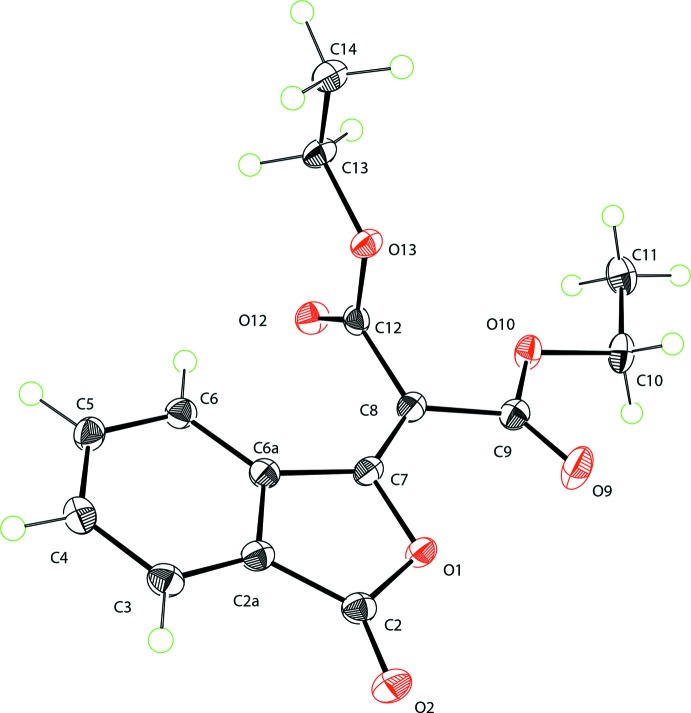
The mol­ecular structure of (**I**), showing displacement ellipsoids drawn at the 50% probability level.

**Figure 2:**
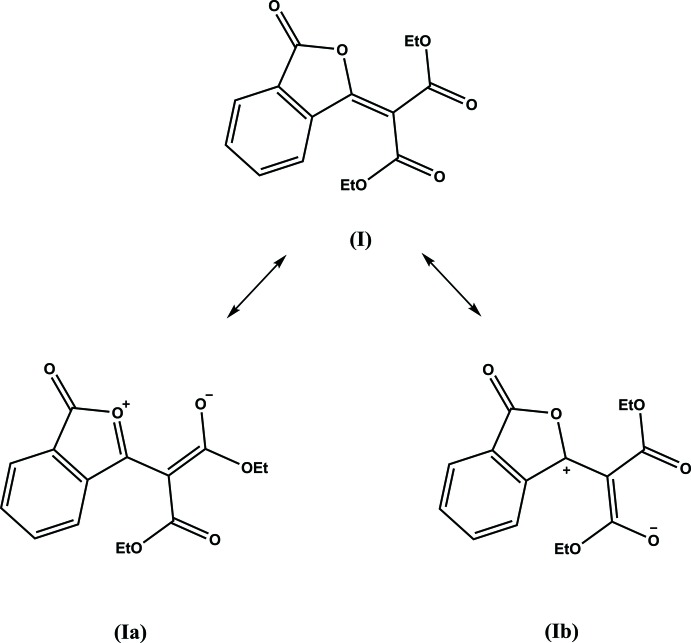
Chemical diagram of a mol­ecule (**I**) and its possible resonance forms (**Ia**) and (**Ib**).

Compound (**I**) crystallizes with one mol­ecule in the asymmetric unit (Fig. 1 ▸). Atoms C8, C9, C10, C11, C12, O9 and O10 are almost coplanar with the isobenzo­furan unit (r.m.s. deviation = 0.024 Å), as shown by the dihedral angle of 5.45 (3)° between these groupings. This mean plane is inter­cepted nearly perpendicularly by the mean plane of the other ester group (O12, C12, O13, C13, and C14), with a dihedral angle of 83.30 (3)° and the torsion angles C9—C8—C12—O12 and C9—C8—C12—O13 of 90.2 (1)° and −89.6 (1)°, respectively. The bond lengths in the carbocyclic ring of the isobenzo­furan unit range from 1.386 (2) Å to 1.398 (2) Å. In the heterocyclic furan ring the distances C2—C2*A* = 1.469 (2) Å and C6*A*—C7 = 1.472 (2) Å are very similar, while the C2—O1 and C7—O1 distances are significantly different. The C2—O1 bond distance of 1.394 (1) Å perfectly matches the corresponding distances in phthalic anhydride [1.396 (5) Å and 1.393 (6) Å (Bates & Cutler, 1977 ▸)]. The shorter C7—O1 distance of 1.385 (1) Å strongly suggests that the bond between the endocyclic oxygen atom O1 and the non-carbonyl carbon atom C7 has an order greater than 1. In the diethyl malonate fragment the distances C8—C9 [1.489 (2) Å] and C8—C12 [1.507 (1) Å] are different most likely due to the particular conformation adopted by the mol­ecule. The bond lengths for the atoms, associated with both the furan ring of the isobenzo­furan unit and the diethyl malonate fragment, indicate that the dipolar resonance form (**Ia**) of (**I**) makes a considerable contribution to its overall mol­ecular electronic structure (Fig. 2 ▸).

According to the structure of the resonance form (**Ia**) a partial positive charge is localized on the oxygen atom of the heterocyclic furan ring, and one of the carbonyl oxygen atoms of the diethyl malonate fragment carries a partial negative charge, which should lead to an electrostatic attraction of these two oxygen atoms. In the structure of (**I**) the O1 and O9 atoms are nearly coplanar (the torsion angles C7—C8—C9—O9 and C9—C8—C7—O1 equal to −10.6 (2)° and 0.7 (2)°, respectively), and the distance O1⋯O9 is 2.756 (2) Å. It can be argued that simple electrostatic attraction is responsible for this close contact.

## Supra­molecular features   

**Figure 3:**
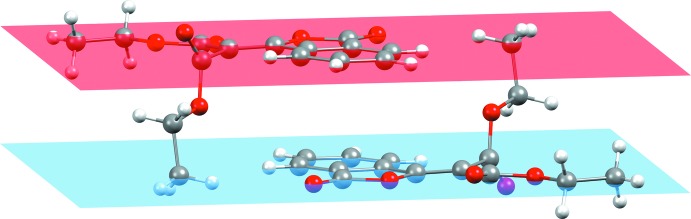
Mol­ecules of (**I**) inter­acting *via* parallel-displaced π–π stacking.

The possible non van der Waals contact in the crystal of (**I**) is a very weak C—H⋯O inter­action (Table 1 ▸), which links the mol­ecules into [100] *C*(8) chains. A parallel-displaced π–π stacking inter­action between mol­ecules of (**I**) is observed with an inter­planar distance of 3.423 Å (Fig. 3 ▸) and inter­molecular furan–benzene and benzene–benzene centroid-to-centroid distances of 3.5379 (13) and 3.7859 (14) Å, respectively.

## Database survey   

In the structure of 3-(3-oxo-1,3-di­hydro­isobenzo­furan-1-yl­idene)pentane-2,4-dione (HIFQUJ; Portilla *et al.*, 2007 ▸) the dominant resonance form resembles (**Ib**). No contact was observed between the endocyclic oxygen atom and the carbonyl oxygen atom of the acetyl group (the distance between these atoms exceeds 4 Å). In 2-meth­oxy­ethyl 3-oxo-2-(3-oxo-2-benzo­furan-1(3*H*)-yl­idene)butano­ate (UBAVIE; Mkrtchyan *et al.*, 2011 ▸), no close contacts exist between the endocyclic oxygen atom and any other oxygen atoms.

In methyl 4,4-dimethyl-3-oxo-2-(3-oxo-2-benzo­furan-1(3*H*)-yl­idene)penta­noate (UBAVEA; Mkrtchyan *et al.*, 2011 ▸), neither of the two carbonyl oxygen atoms of the methyl 4,4-dimethyl-3-oxo­penta­noate fragment are within the same plane as the isobenzo­furan unit. The shortest inter­molecular O⋯O contact is 3.161 Å, which occurs between the endocyclic oxygen atom and that carbonyl oxygen atom, which is closest to the plane of the isobenzo­furan unit. The torsion angle O4—C15—C9—C8 in UBAVEA is 26.81°, while the corresponding torsion angle in (**I**), C7—C8—C9—O9, is 10.6 (2)°.

## Synthesis and crystallization   

The title compound was prepared by the reaction of diethyl malonate with phthaloyl chloride in aceto­nitrile in the presence of tri­ethyl­amine and magnesium chloride (Rathke & Cowan, 1985 ▸). The reagents for the synthesis were purchased from Aldrich and were used as supplied. The crude product was washed with petroleum ether on filter paper and recrystallized from cyclo­hexane solution as colorless crystals (69% yield); m.p. 345–346 K. ^1^H NMR (400 MHz, CDCl_3_) δ 1.38 (*m*, 6H), 4.39 (*m*, 4H), 7.72 (*t*, *J* = 6.7 Hz, 1H), 7.80 (*t*, *J* = 7.4 Hz, 1H), 7.99 (*d*, *J* = 7.8 Hz, 1H), 8.65 (*d*, *J* = 8.2 Hz, 1H). ^13^C NMR (400 MHz, CDCl_3_) δ 135.36; 132.93; 127.58; 126.11; 125.84; 62.18; 62.06; 14.04; 14.01. Found: C, 62.08; H, 4.94%. C_15_H_14_O_6_ Theoretical: C, 62.07; H, 4.86%. The crystalline product was found to be stable to air, water and brief exposure to 1 *M* hydro­chloric acid.

## Refinement   

Crystal data, data collection and structure refinement details are summarized in Table 2 ▸. H atoms were refined using the riding model with C—H = 0.95–0.99 Å and *U*_iso_(H) = 1.2*U*_eq_(C).

## Supplementary Material

Crystal structure: contains datablock(s) I. DOI: 10.1107/S2056989017013962/hb7700sup1.cif

Structure factors: contains datablock(s) I. DOI: 10.1107/S2056989017013962/hb7700Isup3.hkl

Supporting information file. DOI: 10.1107/S2056989017013962/hb7700Isup3.cml

CCDC reference: 1543053

Additional supporting information:  crystallographic information; 3D view; checkCIF report

## Figures and Tables

**Table 1:** Hydrogen-bond geometry (Å, °)

*D*—H⋯*A*	*D*—H	H⋯*A*	*D*⋯*A*	*D*—H⋯*A*
C5—H5⋯O9^i^	0.95	2.48	3.0689 (18)	120

Symmetry code: (i) 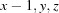.

**Table 2:** Experimental details

Crystal data	
Chemical formula	C_15_H_14_O_6_
*M*_r_	290.27
Crystal system, space group	Triclinic, *P* 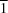
Temperature (K)	93
*a*, *b*, *c* (Å)	7.942 (2), 9.453 (3), 10.226 (2)
α, β, γ (°)	67.706 (15), 72.228 (16), 86.41 (2)
*V* (Å^3^)	675.2 (3)
*Z*	2
Radiation type	Mo *K*α
μ (mm^−1^)	0.11
Crystal size (mm)	0.30 × 0.20 × 0.15

Data collection	
Diffractometer	Rigaku XtaLAB P200
Absorption correction	Multi-scan (*REQAB*; Rigaku, 1998 ▸)
*T*_min_, *T*_max_	0.829, 0.983
No. of measured, independent and observed [*F* ^2^ > 2.0σ(*F* ^2^)] reflections	9890, 2466, 2278
*R*_int_	0.031
(sin θ/λ)_max_ (Å^−1^)	0.602

Refinement	
*R*[*F* ^2^ > 2σ(*F* ^2^)], *wR*(*F* ^2^), *S*	0.028, 0.078, 1.07
No. of reflections	2466
No. of parameters	192
H-atom treatment	H-atom parameters constrained
Δρ_max_, Δρ_min_ (e Å^−3^)	0.25, −0.18

Computer programs: *CrystalClear-SM Expert* (Rigaku, 2014 ▸), *SIR2011* (Burla *et al.*, 2012 ▸), *SHELXL2013* (Sheldrick, 2008 ▸) and *CrystalStructure* (Rigaku, 2014).

## supplementary crystallographic information

### Crystal data

**Table:**

C_15_H_14_O_6_	*Z* = 2
*M**_r_* = 290.27	*F*(000) = 304.00
Triclinic, *P*1	*D*_x_ = 1.428 Mg m^−^^3^
*a* = 7.942 (2) Å	Mo *K*α radiation, λ = 0.71075 Å
*b* = 9.453 (3) Å	Cell parameters from 2290 reflections
*c* = 10.226 (2) Å	θ = 2.3–27.5°
α = 67.706 (15)°	µ = 0.11 mm^−^^1^
β = 72.228 (16)°	*T* = 93 K
γ = 86.41 (2)°	Prism, colorless
*V* = 675.2 (3) Å^3^	0.30 × 0.20 × 0.15 mm

### Data collection

**Table:**

Rigaku XtaLAB P200 diffractometer	2278 reflections with *F*^2^ > 2.0σ(*F*^2^)
Detector resolution: 5.814 pixels mm^-1^	*R*_int_ = 0.031
ω scans	θ_max_ = 25.3°, θ_min_ = 2.3°
Absorption correction: multi-scan (*REQAB*; Rigaku, 1998)	*h* = −9→9
*T*_min_ = 0.829, *T*_max_ = 0.983	*k* = −11→11
9890 measured reflections	*l* = −12→12
2466 independent reflections	

### Refinement

**Table:**

Refinement on *F*^2^	Secondary atom site location: difference Fourier map
*R*[*F*^2^ > 2σ(*F*^2^)] = 0.028	Hydrogen site location: inferred from neighbouring sites
*wR*(*F*^2^) = 0.078	H-atom parameters constrained
*S* = 1.07	*w* = 1/[σ^2^(*F*_o_^2^) + (0.0425*P*)^2^ + 0.0953*P*] where *P* = (*F*_o_^2^ + 2*F*_c_^2^)/3
2466 reflections	(Δ/σ)_max_ < 0.001
192 parameters	Δρ_max_ = 0.25 e Å^−^^3^
0 restraints	Δρ_min_ = −0.18 e Å^−^^3^
Primary atom site location: structure-invariant direct methods	

### Special details

**Table:**

Geometry. All esds (except the esd in the dihedral angle between two l.s. planes) are estimated using the full covariance matrix. The cell esds are taken into account individually in the estimation of esds in distances, angles and torsion angles; correlations between esds in cell parameters are only used when they are defined by crystal symmetry. An approximate (isotropic) treatment of cell esds is used for estimating esds involving l.s. planes.
Refinement. Refinement was performed using all reflections. The weighted R-factor (wR) and goodness of fit (S) are based on F^2^. R-factor (gt) are based on F. The threshold expression of F^2^ > 2.0 sigma(F^2^) is used only for calculating R-factor (gt).

### Fractional atomic coordinates and isotropic or equivalent isotropic displacement parameters (Å^2^)

**Table:**

	*x*	*y*	*z*	*U*_iso_*/*U*_eq_	
O1	0.78603 (9)	0.26223 (8)	−0.08336 (8)	0.01823 (18)	
O2	0.88520 (10)	0.18829 (9)	−0.27766 (9)	0.0250 (2)	
O9	0.92060 (10)	0.33329 (10)	0.10424 (10)	0.0292 (2)	
O10	0.71494 (9)	0.40999 (9)	0.26677 (8)	0.02015 (19)	
O12	0.34235 (10)	0.41829 (8)	0.23018 (8)	0.02202 (19)	
O13	0.39408 (9)	0.17091 (8)	0.34225 (8)	0.01784 (18)	
C2	0.76082 (14)	0.20746 (12)	−0.18525 (12)	0.0188 (2)	
C2A	0.56886 (14)	0.18300 (12)	−0.15155 (12)	0.0176 (2)	
C3	0.47681 (14)	0.12685 (12)	−0.21879 (12)	0.0203 (2)	
H3	0.5367	0.0970	−0.2992	0.024*	
C4	0.29321 (14)	0.11623 (12)	−0.16343 (12)	0.0209 (2)	
H4	0.2255	0.0764	−0.2053	0.025*	
C5	0.20674 (14)	0.16332 (12)	−0.04692 (12)	0.0199 (2)	
H5	0.0809	0.1570	−0.0126	0.024*	
C6	0.30017 (13)	0.21919 (12)	0.02009 (12)	0.0181 (2)	
H6	0.2406	0.2513	0.0990	0.022*	
C6A	0.48425 (14)	0.22634 (11)	−0.03286 (11)	0.0164 (2)	
C7	0.62379 (13)	0.27243 (11)	0.01279 (11)	0.0164 (2)	
C8	0.61249 (13)	0.31329 (11)	0.12678 (12)	0.0170 (2)	
C9	0.76891 (13)	0.35264 (12)	0.16046 (12)	0.0182 (2)	
C10	0.85459 (14)	0.45643 (13)	0.30998 (13)	0.0233 (3)	
H10A	0.9277	0.5452	0.2271	0.028*	
H10B	0.9323	0.3712	0.3371	0.028*	
C11	0.76446 (15)	0.49876 (14)	0.44073 (13)	0.0267 (3)	
H11A	0.6954	0.4091	0.5228	0.032*	
H11B	0.6853	0.5810	0.4131	0.032*	
H11C	0.8537	0.5338	0.4718	0.032*	
C12	0.43443 (13)	0.31193 (12)	0.23574 (11)	0.0162 (2)	
C13	0.22714 (13)	0.15072 (12)	0.46014 (12)	0.0202 (2)	
H13A	0.1283	0.1846	0.4175	0.024*	
H13B	0.2327	0.2116	0.5192	0.024*	
C14	0.19989 (14)	−0.01740 (12)	0.55625 (12)	0.0220 (2)	
H14A	0.1880	−0.0757	0.4978	0.026*	
H14B	0.0921	−0.0353	0.6404	0.026*	
H14C	0.3018	−0.0506	0.5931	0.026*	

### Atomic displacement parameters (Å^2^)

**Table:**

	*U*^11^	*U*^22^	*U*^33^	*U*^12^	*U*^13^	*U*^23^
O1	0.0135 (4)	0.0209 (4)	0.0182 (4)	0.0008 (3)	−0.0019 (3)	−0.0076 (3)
O2	0.0191 (4)	0.0308 (5)	0.0227 (4)	0.0033 (3)	−0.0006 (3)	−0.0122 (4)
O9	0.0152 (4)	0.0410 (5)	0.0386 (5)	0.0039 (3)	−0.0071 (4)	−0.0241 (4)
O10	0.0160 (4)	0.0236 (4)	0.0251 (4)	0.0010 (3)	−0.0078 (3)	−0.0125 (3)
O12	0.0203 (4)	0.0195 (4)	0.0236 (4)	0.0050 (3)	−0.0050 (3)	−0.0073 (3)
O13	0.0145 (4)	0.0173 (4)	0.0184 (4)	0.0013 (3)	−0.0019 (3)	−0.0056 (3)
C2	0.0194 (5)	0.0169 (5)	0.0174 (5)	0.0022 (4)	−0.0042 (4)	−0.0048 (4)
C2A	0.0175 (5)	0.0155 (5)	0.0167 (5)	0.0024 (4)	−0.0038 (4)	−0.0040 (4)
C3	0.0233 (6)	0.0199 (5)	0.0179 (5)	0.0036 (4)	−0.0061 (4)	−0.0080 (4)
C4	0.0226 (5)	0.0202 (6)	0.0220 (6)	0.0018 (4)	−0.0103 (5)	−0.0075 (5)
C5	0.0165 (5)	0.0203 (5)	0.0215 (6)	0.0022 (4)	−0.0066 (4)	−0.0060 (4)
C6	0.0171 (5)	0.0188 (5)	0.0174 (5)	0.0027 (4)	−0.0044 (4)	−0.0066 (4)
C6A	0.0174 (5)	0.0136 (5)	0.0162 (5)	0.0015 (4)	−0.0055 (4)	−0.0033 (4)
C7	0.0134 (5)	0.0140 (5)	0.0176 (5)	0.0012 (4)	−0.0028 (4)	−0.0032 (4)
C8	0.0153 (5)	0.0146 (5)	0.0188 (5)	0.0012 (4)	−0.0047 (4)	−0.0043 (4)
C9	0.0173 (5)	0.0157 (5)	0.0201 (5)	0.0007 (4)	−0.0051 (4)	−0.0055 (4)
C10	0.0190 (5)	0.0230 (6)	0.0338 (7)	0.0014 (4)	−0.0136 (5)	−0.0126 (5)
C11	0.0286 (6)	0.0274 (6)	0.0314 (7)	0.0030 (5)	−0.0162 (5)	−0.0137 (5)
C12	0.0164 (5)	0.0176 (5)	0.0169 (5)	0.0001 (4)	−0.0071 (4)	−0.0073 (4)
C13	0.0153 (5)	0.0220 (6)	0.0198 (6)	0.0009 (4)	−0.0003 (4)	−0.0081 (5)
C14	0.0188 (5)	0.0213 (6)	0.0217 (6)	−0.0005 (4)	−0.0023 (4)	−0.0064 (5)

### Geometric parameters (Å, º)

**Table:**

O1—C7	1.3854 (12)	C6—C6A	1.3916 (15)
O1—C2	1.3940 (13)	C6—H6	0.9500
O2—C2	1.1997 (13)	C6A—C7	1.4719 (14)
O9—C9	1.2019 (13)	C7—C8	1.3377 (15)
O10—C9	1.3391 (13)	C8—C9	1.4888 (14)
O10—C10	1.4579 (13)	C8—C12	1.5070 (14)
O12—C12	1.1995 (13)	C10—C11	1.4986 (16)
O13—C12	1.3435 (13)	C10—H10A	0.9900
O13—C13	1.4591 (12)	C10—H10B	0.9900
C2—C2A	1.4695 (15)	C11—H11A	0.9800
C2A—C3	1.3862 (15)	C11—H11B	0.9800
C2A—C6A	1.3919 (15)	C11—H11C	0.9800
C3—C4	1.3886 (16)	C13—C14	1.5055 (16)
C3—H3	0.9500	C13—H13A	0.9900
C4—C5	1.3978 (16)	C13—H13B	0.9900
C4—H4	0.9500	C14—H14A	0.9800
C5—C6	1.3904 (15)	C14—H14B	0.9800
C5—H5	0.9500	C14—H14C	0.9800
			
C7—O1—C2	109.90 (8)	O9—C9—O10	124.58 (10)
C9—O10—C10	115.85 (8)	O9—C9—C8	125.85 (10)
C12—O13—C13	116.01 (8)	O10—C9—C8	109.55 (9)
O2—C2—O1	120.62 (10)	O10—C10—C11	106.67 (9)
O2—C2—C2A	132.18 (11)	O10—C10—H10A	110.4
O1—C2—C2A	107.20 (9)	C11—C10—H10A	110.4
C3—C2A—C6A	122.56 (10)	O10—C10—H10B	110.4
C3—C2A—C2	129.50 (10)	C11—C10—H10B	110.4
C6A—C2A—C2	107.95 (9)	H10A—C10—H10B	108.6
C2A—C3—C4	117.04 (10)	C10—C11—H11A	109.5
C2A—C3—H3	121.5	C10—C11—H11B	109.5
C4—C3—H3	121.5	H11A—C11—H11B	109.5
C3—C4—C5	120.88 (10)	C10—C11—H11C	109.5
C3—C4—H4	119.6	H11A—C11—H11C	109.5
C5—C4—H4	119.6	H11B—C11—H11C	109.5
C6—C5—C4	121.67 (10)	O12—C12—O13	124.83 (10)
C6—C5—H5	119.2	O12—C12—C8	126.32 (10)
C4—C5—H5	119.2	O13—C12—C8	108.85 (8)
C5—C6—C6A	117.49 (10)	O13—C13—C14	106.78 (8)
C5—C6—H6	121.3	O13—C13—H13A	110.4
C6A—C6—H6	121.3	C14—C13—H13A	110.4
C6—C6A—C2A	120.32 (10)	O13—C13—H13B	110.4
C6—C6A—C7	132.70 (10)	C14—C13—H13B	110.4
C2A—C6A—C7	106.98 (9)	H13A—C13—H13B	108.6
C8—C7—O1	121.51 (9)	C13—C14—H14A	109.5
C8—C7—C6A	130.55 (10)	C13—C14—H14B	109.5
O1—C7—C6A	107.90 (9)	H14A—C14—H14B	109.5
C7—C8—C9	123.84 (9)	C13—C14—H14C	109.5
C7—C8—C12	120.08 (9)	H14A—C14—H14C	109.5
C9—C8—C12	115.97 (9)	H14B—C14—H14C	109.5
			
C7—O1—C2—O2	−179.09 (9)	C6—C6A—C7—O1	−177.86 (10)
C7—O1—C2—C2A	0.60 (11)	C2A—C6A—C7—O1	2.72 (11)
O2—C2—C2A—C3	1.1 (2)	O1—C7—C8—C9	0.65 (16)
O1—C2—C2A—C3	−178.50 (10)	C6A—C7—C8—C9	178.04 (9)
O2—C2—C2A—C6A	−179.21 (11)	O1—C7—C8—C12	−175.47 (8)
O1—C2—C2A—C6A	1.15 (11)	C6A—C7—C8—C12	1.92 (17)
C6A—C2A—C3—C4	0.54 (15)	C10—O10—C9—O9	2.55 (15)
C2—C2A—C3—C4	−179.85 (10)	C10—O10—C9—C8	−179.01 (8)
C2A—C3—C4—C5	1.21 (15)	C7—C8—C9—O9	−10.62 (17)
C3—C4—C5—C6	−1.41 (16)	C12—C8—C9—O9	165.65 (10)
C4—C5—C6—C6A	−0.18 (15)	C7—C8—C9—O10	170.96 (9)
C5—C6—C6A—C2A	1.90 (15)	C12—C8—C9—O10	−12.77 (12)
C5—C6—C6A—C7	−177.45 (10)	C9—O10—C10—C11	−173.45 (9)
C3—C2A—C6A—C6	−2.15 (15)	C13—O13—C12—O12	−1.65 (14)
C2—C2A—C6A—C6	178.17 (9)	C13—O13—C12—C8	178.17 (8)
C3—C2A—C6A—C7	177.35 (9)	C7—C8—C12—O12	−93.36 (14)
C2—C2A—C6A—C7	−2.32 (11)	C9—C8—C12—O12	90.22 (13)
C2—O1—C7—C8	175.88 (9)	C7—C8—C12—O13	86.82 (11)
C2—O1—C7—C6A	−2.03 (10)	C9—C8—C12—O13	−89.60 (10)
C6—C6A—C7—C8	4.48 (19)	C12—O13—C13—C14	173.90 (8)
C2A—C6A—C7—C8	−174.94 (11)		

### Hydrogen-bond geometry (Å, º)

**Table:**

*D*—H···*A*	*D*—H	H···*A*	*D*···*A*	*D*—H···*A*
C5—H5···O9^i^	0.95	2.48	3.0689 (18)	120

Symmetry code: (i) *x*−1, *y*, *z*.
